# Preparation of an In-House Reference Material Containing Fumonisins in Thai Rice and Matrix Extension of the Analytical Method for Japanese Rice

**DOI:** 10.3390/toxins1020188

**Published:** 2009-12-08

**Authors:** Norhafniza Awaludin, Reiko Nagata, Tomomi Kawasaki, Masayo Kushiro

**Affiliations:** 1 Biotechnology Research Centre, Malaysian Agricultural Research and Development Institute, GPO, P.O. Box 12301, 50774 Kuala Lumpur, Malaysia; Email: hafniza@mardi.my (N.A.); 2 National Food Research Institute, National Agriculture and Food Research Organization, 2-1-12 Kannondai, Tsukuba 305-8642, Japan; Email: nagatar@affrc.go.jp (R.N.); shirotan@affrc.go.jp (T.K.)

**Keywords:** reference material, *Fusarium*, fumonisin, Thai-rice, HPLC-FL

## Abstract

Mycotoxin contamination in rice is less reported, compared to that in wheat or maize, however, some *Fusarium* fungi occasionally infect rice in the paddy field. Fumonisins are mycotoxins mainly produced by *Fusarium verticillioides*, which often ruins maize. Rice adherent fungus *Gibberella fujikuroi* is taxonomically near to *F. verticillioides*, and there are sporadic reports of fumonisin contamination in rice from Asia, Europe and the United States. Therefore, there exists the potential risk of fumonisin contamination in rice as well as the need for the validated analytical method for fumonisins in rice. Although both natural and spiked reference materials are available for some *Fusarium* mycotoxins in matrices of wheat and maize, there are no reference materials for *Fusarium* mycotoxins in rice. In this study, we have developed a method for the preparation of a reference material containing fumonisins in Thai rice. A ShakeMaster grinding machine was used for the preparation of a mixed material of blank Thai rice and *F. verticillioides*-infected Thai rice. The homogeneity of the mixed material was confirmed by one-way analysis of variance, which led this material to serve as an in-house reference material. Using this reference material, several procedures to extract fumonisins from Thai rice were compared. Accordingly, we proved the applicability of an effective extraction procedure for the determination of fumonisins in Japanese rice.

## Abbreviations

ppm: parts par million
RM: reference material
FB1: fumonisin B1
FB2: fumonisin B2
SPE: solid phase extraction
HPLC-FL: high performance liquid chromatography with fluorescence detection

## 1. Introduction

Rice is one of the major staples, feeding about half the world's population [1]. Rice is of tropical origin and is generally considered to be tolerant to fungi, compared to wheat and maize; however, some *Fusarium* fungi occasionally infect rice in the paddy field [2]. Rice is susceptible to a *Fusarium* fungus *Fusarium/Gibberella fujikuroi*, which causes disorders in rice plants with a symptom of unusually higher height.

Fumonisins are newly found *Fusarium* mycotoxins first identified in 1988 and are among the most important mycotoxins with regards to food and feed safety [3,4]. The most abundant analogue in nature is fumonisin B1 (FB1), followed by fumonisin B2 (FB2) [5]. Fumonisins are produced mainly by *Fusarium verticillioides*, a maize adherent fungus, and fumonisin contamination in maize has been observed worldwide [6]. FB1 has been implicated with various disorders in animals such as leukoencephalomalacia in horses, pulmonary oedema syndrome in pigs, showing nephrotoxicity, hepatotoxicity, and hepatocellular carcinogenicity in rats [7]. Concerning the human toxicity, the International Agency for Research on Cancer (IARC) evaluated the FB1 derived from *F. verticillioides* as Group 2B, i.e. a possible human carcinogen, in 2002 [8]. Fumonisins have also been receiving attention as neurotoxins because they have been found to be a potential cause of neural tube defects in human babies since first reported in 1999 among a high maize-consuming population in the United States (Texas)-Mexico border area [9]. In European Union, the regulatory limits from 0.2 to 4 parts per million (ppm) have set, depending on the type of food.

So far maize and maize-based products are the only commodities which are known to contain significant amounts of fumonisins. However, considering the fact that rice adherent fungus *G. fujikuroi* is taxonomically near to *F. verticillioides* (formerly *F. moniliforme*), there exists a potential risk of fumonisin contamination in rice [10,11,12]. Furthermore, there are sporadic reports of low-level contamination of fumonisins in rice [13,14]: While the contents of fumonisins in maize sometimes reach above 10 ppm, those in rice are around or below 1 ppm or their pollutions are usually opportunistic. Therefore, there is a need for the establishment of a validated method for the determination of fumonisins in rice at a lower level.

The difficulty of obtaining accurate analytical data on the concentrations of contaminants can be mitigated if reference materials (RMs) with known concentrations of contaminants in certain matrices of the object are available. Currently, both natural and spiked RMs are available for some *Fusarium* mycotoxins such as deoxynivalenol in matrices of wheat and maize [15]. On the other hand, there are no RMs for *Fusarium* mycotoxins in rice, including a reference material (RM) of rice containing fumonisins.

Recently, a ShakeMaster grinding machine was used to prepare an in-house RM of Japanese rice (a cultivar of short grain) containing sterigmatocystin. By mixing blank Japanese rice and artificially *Aspergillus versicolor*-infected Japanese rice, an in-house RM of 3 ppm level was successfully obtained [16]. In this study, we prepared an in-house RM containing fumonisins in the matrix of Thai rice, one of the major rice cultivars of long grain, which feeds lots of population in the world. The homogeneity of the mixed material was confirmed by one-way analysis of variance at lower level, which led this material to serve as an in-house RM for fumonisins in Thai rice at lower level (<1 ppm). Using this RM, we assessed the applicability of a method for the analysis of fumonisins in Japanese rice using high performance liquid chromatography with fluorescence detection (HPLC-FL).

## 2. Results and Discussion

### 2.1. Preparation of an in-house RM of Thai rice containing low-level of fumonisins

The need of RMs for the development of analytical methods has long been discussed, because spike and recovery tests do not reflect the natural binding of matrices or major elements in living organisms (protein, carbohydrates, and fats) and trace elements or contaminants at ppm level. In spite of the potential risk of fumonisin contamination in rice, no RM of rice containing fumonisins is commercially available. In this study, the applicability of the ShakeMaster grinding machine, recently used for the preparation of in-house RM of rice containing sterigmatocystin at 3 ppm level [16], was examined. About 40 g of Thai rice culture was diluted with blank Thai rice in a ShakeMaster machine until 2,000 g of mixed material of Thai rice containing FB1 at 0.1-1.0 ppm level was obtained. The values for FB1 concentration of randomly picked up from this mixed material were sufficient to pass the homogeneity test (Table 1). Therefore, the method of mixing and grinding in a ShakeMaster machine was proved to be applicable for the preparation of 2,000 g of an in-house RM containing fumonisins at lower level (<1 ppm) in the matrix of Thai rice. In case of preparation for larger scale, more efficient system which enables the grinding of 1 kg of material at once might be required.

**Table 1:** Homogeneity test of FB1 of artificially prepared fumonisin contaminated Thai rice.

	FB1 concentration (ppm)								
Sample ID	1	2		3	4		5	6	7
Replicate 1	0.107	0.134		0.119	0.151		0.109	0.139	0.0939
Replicate 2	0.148	0.0823		0.115	0.140		0.123	0.128	0.106
	**One-way ANOVA table**								
Source	Sum of squares		Degree of freedom			Mean square		*F-value*	*p-value*
between	0.002895847		6			0.000482641		1.36386	0.34427
within	0.002477150		7			0.000353879			
total	0.005372997		13						
	***p-value* > 0.05 Accept**								

### 2.2. Extraction and purification procedures

Values of FB1 and FB2 in the in-house RM were compared for different extraction volume and submergence periods, as shown in Table 2 and Table 3. For the extraction of fumonisins from food samples, two-fold of sample volume of methanol-water (3 + 1, v/v) (for example, 100 mL of methanol-water (3 + 1, v/v) to 50 g of corn) has often been used as extraction procedure in the past since the ratio was adopted by AOAC Official Method 995.15, a validated method for the analysis of fumonisins in corn [17]. However, in our previous study this extraction ratio (two-fold of sample volume) was not sufficient for fumonisins in Japanese rice and a five-fold increase of further 2.5-fold increase of solvent volume (for example, 50 mL of methanol-water (3 + 1, v/v) to 10 g of Japanese rice) was required [18]. In the Thai rice matrix, we compared six-fold, eight-fold and ten-fold solvent for extraction. As shown in Table 2, significant higher values of FB1 and FB2 were obtained in eight-fold solvent volume (80 mL) than in six-fold solvent volume (60 mL), while no significant difference was observed between eight-fold solvent volume (80 mL) and ten-fold solvent volume (100 mL). We adopted another extraction technique using submergence in water prior to the addition of methanol, which has been effective in the extraction of fumonisins in Japanese rice [18]. The technique was also proved to be effective and showed dramatic increase in the values of FB1 and FB2 in the matrix of Thai rice (Table 3).

**Figure 1:**
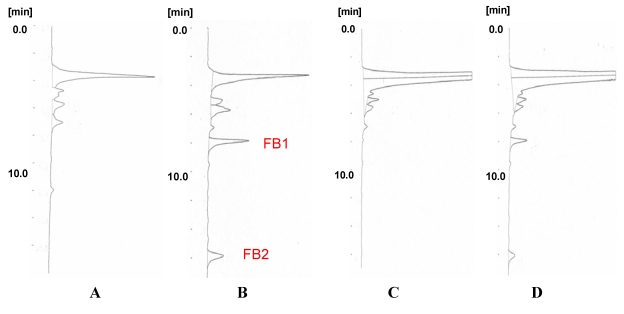
HPLC-FL chromatogram of fumonisins. A: Blank solution. B: Standard solution of FB1 and FB2. C: The purified extract of 0 min submerged Thai rice (spiked FB1 and FB2 at 0.2 ppm level). D: The purified extract of 30 min submerged Thai rice (spiked FB1 and FB2 at 0.2 ppm level).

The recoveries of FB1 and FB2 spiked to blank Thai rice are shown in Table 4. Spike and recovery test was done in triplicate at 0.2 ppm-spiked level and the average value was shown with standard deviation (S.D.). Considering the result of Table 2, the eight-fold volume of extraction solvent was adopted and the effect of submergence was evaluated. As shown, submergence was also worked and resulted in low S.D. for repeatability (1-2%) and proper recovery (62-67%) (Table 4: 30 min submergence) (Figure 1D). On the other hand, without submergence, the value of FB1 was under 0.06 ppm which means the recovery was below 30% (Table 4: 0 min submergence) (Figure 1C). For the clean-up step for fumonisins, a kind of solid phase extraction (SPE) cartridge of strong anion exchange (SAX), Bond Elut SAX, was used followed by AOAC Official Method 995.15 [17], and it is proved to work well. 

**Table 2:** Effect of extraction volume on the values of fumonisins in in-house RM.

Extraction solvent volume	FB1 concentration (ppm)(n = 3)	FB2 concentration (ppm)(n = 3)
60 mL	0.14 ± 0.006^a^	0.061 ± 0.001^a^
80 mL	0.18 ± 0.006^b^	0.087 ± 0.006^b^
100 mL	0.16 ± 0.001^ab^	0.076 ± 0.004^b^

a, b Mean values which do not share superscript letters in the corresponding columns were significantly different (*P* < 0.05).

**Table 3:** Effect of submergence period on the values of fumonisins in in-house RM.

Submergence period	FB1 concentration (ppm)(n = 3)	FB2 concentration (ppm)(n = 3)
0 min	0.17 ± 0.006	0.081 ± 0.003
30 min	0.32 ± 0.01*	0.17 ± 0.005*

*Mean values in the corresponding columns were significantly different (*P* < 0.05).

**Table 4:** Effect of submergence on the recovery of fumonisins from 0.2 ppm spiked sample.

Submergence period	FB1 recovery (%)(n = 3)	FB2 recovery (%)(n = 3)
0 min	<LOQ	<LOQ
30 min	62 ± 2	67 ± 1

<LOQ = below limit of quantification (0.06 ppm).

## 3. Experimental Section

### 3.1. Samples and chemicals

Thai rice harvested in the 2006 was obtained from a retail shop and stored at 4 °C until used as a sample. Standard FB1 and FB2 were purchased from Wako Chemicals (Osaka, Japan). All other reagents were of HPLC grade. Stock solutions (100 μg/mL each) were prepared in acetonitrile-H_2_O solution (1 + 1, v/v) as recommended by AOAC Official Method 995.15 [17], and were diluted to the desired concentration with acetonitrile-H_2_O solution. Fumonisin stocks and standard working solutions were kept at 4 °C up to 6 months.

### 3.2. Preparation of artificially fumonisin-contaminated Thai rice

Wild type *Fusarium verticillioides* MAFF239106 (Genebank, National Institute of Agrobiological Resources, Ministry of Agriculture, Forestry and Fisheries, Japan) was used for the preparation of artificially molded Thai rice contaminated with fumonisins. The fungus was pre-inoculated and grown on a potato dextrose agar plate for 3 days. Thirty grams of Thai rice wetted with 15 mL of water for three hours was autoclaved and served as a fumonisin producing culture medium. The fungus grown on the plate was sliced as three debris of agar and inoculated on the above medium, and kept for two weeks at 25 °C until to accumulate ca. 50 ppm of fumonisins in the Thai rice culture medium. The culture was ground with blank Thai rice at the ratio of 1:10 in a ShakeMaster (Biomedical Sciences, Tokyo, Japan) equipped with a container of 400 g capacity to make a fine powder. This procedure was repeated to obtain 2,000 g of powdered Thai rice containing low-level of fumonisins (0.1-1.0 ppm). Randomly picked up in-house RM samples of 5.0 g each were extracted in a 50 mL tube with a screw cap using 40 mL methanol-H_2_O solution (3 + 1, v/v) by vigorous vortexing for 1 min with a Vortex Genie2 (Scientific Industries, NY, USA), and the supernatant was purified and analyzed in the procedure described in *3.3*. Homogeneity of artificially prepared fumonisins contaminated Thai rice powder was confirmed by one-way analysis of variance (ANOVA). The powder which passed the homogeneity test as shown in Table 1 was served as an in-house RM.

### 3.3. Extraction and clean-up

Commercial Thai rice (ca. 60 g) was milled for 3 min with a Waring Laboratory Blender (Model 7012S, Waring Commercial, CT, USA) to yield a fine powder which passes through 0.6 mm screen. An accurately weighed 10.0 g of Thai rice powder was extracted in a 300 mL Erlenmeyer flask with a ground-in stopper using 80 mL methanol-H_2_O solution (3+1, v/v) by vigorous shaking on a shaker (Laboratory shaker, TAITEC, Japan) for 60 min. As for extraction solvent, the portion of water (20 mL) and that of methanol (60 mL) was used separately as follows. First, the sample was mixed and submerged with 20 mL of water in a flask with a stopper for 30 min with vortex on a vortex machine (Vortex Genie2) for 30 sec at 10 min. Second, extraction was followed by the addition of 60 mL of methanol. The extract was purified by a SPE cartridge (Bond Elut SAX, Varian, CA, USA). An accurate volume of filtrate (8.0 mL of Thai rice extract) was loaded onto the SAX cartridge and washed with 3 mL of methanol-water (3 + 1, v/v) followed by 3 mL of methanol. Fumonisins were eluted with 7 mL 1% methanolic acetic acid at a flow rate less than 1 mL/min. The eluate was collected in a 10 mL amber glass tube and evaporated at 60 °C under a gentle flow of nitrogen. The tube was rinsed with 1 mL methanol and evaporate additional methanol to dryness to ensure that remain of acetic acid has evaporated. The residue was redissolved in 1.0 mL acetonitrile-water (1 + 1, v/v) and served as the sample solution for automatic injection to HPLC-FL with pre-column derivatization as previously reported [18].

### 3.4. Spike and recovery test

For the recovery test of spiked sample, standard fumonisins solution (0.8 mL of 2.5 μg/mL FB1 and FB2 mixture to make 0.2 ppm (=mg/kg) spiked samples) were added 5 minutes prior to the addition of extraction solvent, and followed by the extraction and cleanup procedure as described above. Recovery was calculated by the equation as follows: 

Recovery (%) = (A - B)/A × D × 100

where A = signal of spiked sample, B = signal of blank sample, D = dilution factor (=5.0 or 10.0 (g)/40 or 80 (mL) × 8.0 (mL)/1.0 (mL) = 1.0). As mentioned in *3.3*, we added water and methanol for extraction separately. The sum of 20 mL water and 60 mL methanol becomes a little less than 80 mL (ca. 78 mL), which substantially treated as 80 mL. 

### 3.5. Apparatus and determination condition for HPLC-FL analysis

HPLC-FL analysis was performed using an LC-10A series HPLC system (Shimadzu, Kyoto, Japan) equipped with a C18 L-column (250 × 4.6 mm i.d., 5 μm spherical particle size; CERI, Tokyo, Japan), an auto-injector SIL-10A (Shimadzu) and a fluorescence detector RF-10AXL (Shimadzu). The mobile phase was isocratic and composed of methanol-0.1 M sodium phosphate monobasic (77 + 23, v/v), adjusted to pH 3.3 with *o*-phosphoric acid. The mobile phase flow-rate was 1.0 mL/min and column oven temperature was 40 °C. Injection was made by programming with precolumn derivatization so that 10 μL of fluorescent reagent mixture (0.3 M *o*-phthalaldehyde in methanol - 0.1 M disodium tetraborate - 2-mercaptoethanol (1 + 5 + 0.05, v/v) and 10 μL of sample solution was mixed followed by injection of 5 μL. Fluorescence was measured with excitation at 335 nm and emission at 440 nm with a slit width of 12 nm. Chromatographic data was analyzed using Chromatopac system (Shimadzu). Calibration curves were based on the analysis of working standard solution in the ranges of 0.05-1.0 μg/mL (0.05, 0.1, 0.2, 0.5 and 1.0 μg/mL) for FB1 and FB2. LOD was calculated from the standard curve (*r*^2^ > 0.99) and the LOQ was determined as twice the LOQ.

## 4. Conclusions

In this study, we prepared 2,000 g sample of an in-house RM of fumonisins in a Thai rice matrix, which was helpful to assess the effectiveness of extraction and purification procedures developed for the analysis of fumonisins in Japanese rice. For analysis of the distribution of RM, a more efficient method for the preparation as well as the stability test will be required.
